# Antidepressants for the prevention of depression following first-episode psychosis (ADEPP): study protocol for a multi-centre, double-blind, randomised controlled trial

**DOI:** 10.1186/s13063-023-07499-3

**Published:** 2023-10-06

**Authors:** Edward R. Palmer, Siân Lowri Griffiths, Ben Watkins, Tyler Weetman, Ryan Ottridge, Smitaa Patel, Rebecca Woolley, Sarah Tearne, Pui Au, Eleanor Taylor, Zara Sadiq, Hareth Al-Janabi, Barnaby Major, Charlotte Marriott, Nusrat Husain, Mohammad Zia Ul Haq Katshu, Domenico Giacco, Nicholas M. Barnes, James T. R. Walters, Thomas R. E. Barnes, Max Birchwood, Richard Drake, Rachel Upthegrove

**Affiliations:** 1 Institute for Mental Health, School of Psychology, University of Birmingham, Birmingham, UK; 2 Birmingham and Solihull Mental Health Foundation Trust, Birmingham, UK; 3 Early Intervention Service, Birmingham Women’s and Children’s NHS Trust, Birmingham, UK; 4 Birmingham Clinical Trials Unit, University of Birmingham, Birmingham, UK; 5 Health Economics Unit, Institute of Applied Health Research, College of Medical and Dental Sciences, University of Birmingham, Birmingham, UK; 6 Herefordshire and Worcestershire Health and Care NHS Trust, Worcester, UK; 7 Division of Psychology and Mental Health, University of Manchester, Manchester, UK; 8 Mersey Care NHS Foundation Trust, Merseyside, UK; 9 Institute of Mental Health, Division of Mental Health and Neurosciences University of Nottingham, Nottingham, UK; 10 Nottinghamshire Healthcare National Health Service Foundation Trust, Nottingham, UK; 11 Division of Health Sciences, Warwick Medical School, University of Warwick, Warwick, UK; 12 Coventry and Warwickshire Partnership NHS Trust, Coventry, UK; 13 Institute of Clinical Sciences, College of Medical and Dental Sciences, University of Birmingham, Birmingham, UK; 14 Centre for Neuropsychiatric Genetics and Genomics, Division of Psychological Medicine and Clinical Neurosciences, Cardiff University, Cardiff, Wales UK; 15 Division of Psychiatry, Imperial College London, London, UK

**Keywords:** Prevention, Depression, Antidepressant, Functioning, First-episode psychosis

## Abstract

**Background:**

Depressive episodes are common after first-episode psychosis (FEP), affecting more than 40% of people, adding to individual burden, poor outcomes, and healthcare costs. If the risks of developing depression were lower, this could have a beneficial effect on morbidity and mortality, as well as improving outcomes. Sertraline is a selective serotonin reuptake inhibitor and a common first-line medication for the treatment of depression in adults. It has been shown to be safe when co-prescribed with antipsychotic medication, and there is evidence that it is an effective treatment for depression in established schizophrenia. We present a protocol for a multi-centre, double-blind, randomised, placebo-controlled clinical trial called ADEPP that aims to investigate the efficacy and cost-effectiveness of sertraline in preventing depression after FEP.

**Methods:**

The recruitment target is 452 participants between the ages of 18 and 65 years who are within 12 months of treatment initiation for FEP. Having provided informed consent, participants will be randomised to receive either 50 mg of sertraline daily or matched placebo for 6 months, in addition to treatment as usual. The primary outcome measure will be a comparison of the number of new cases of depression between the treatment and placebo arms over the 6-month intervention phase. Secondary outcomes include suicidal behaviour, anxiety, rates of relapse, functional outcome, quality of life, and resource use.

**Discussion:**

The ADEPP trial will test whether the addition of sertraline following FEP is a clinically useful, acceptable, and cost-effective way of improving outcomes following FEP.

**Trial registration:**

ISRCTN12682719 registration date 24/11/2020.

**Supplementary Information:**

The online version contains supplementary material available at 10.1186/s13063-023-07499-3.

## Administrative information

Note: the numbers in curly brackets in this protocol refer to SPIRIT checklist item numbers. The order of the items has been modified to group similar items (see http://www.equator-network.org/reporting-guidelines/spirit-2013-statement-defining-standard-protocol-items-for-clinical-trials/).

**Table:** 

Title {1}	Antidepressant for the prevention of depression following first-episode psychosis (ADEPP): study protocol for a multi-centre, double-blind, randomised, controlled trial.
Trial registration {2a and 2b}.	ISRCTN12682719 registration date 24/11/2020
Protocol version {3}	V11.0, 07/12/2022
Funding {4}	NIHR award NIHR127700 This project is funded by the National Institute for Health Research (NIHR) Health Technology Assessment Programme (project reference 127,700). The Funder of the trial has had no role in the trial design, data collection, data analysis or data interpretation. MB and DG are supported by the NIHR Applied Research Collaboration (ARC) West Midlands. The study also acknowledges support by the NIHR Oxford Health Biomedical Research Centre. The views expressed are those of the author(s) and not necessarily those of the NIHR or the Department of Health and Social Care.
Author details {5a}	^1^Institute for Mental Health, School of Psychology, University of Birmingham, Birmingham, United Kingdom ^2^Birmingham and Solihull Mental Health Foundation Trust, Birmingham, United Kingdom ^3^Early Intervention Service, Birmingham Women’s and Children’s NHS Trust, Birmingham, United Kingdom ^4^Birmingham Clinical Trials Unit, University of Birmingham, Birmingham, United Kingdom ^5^Institute of Applied Health Research, College of Medical and Dental Sciences, University of Birmingham, Birmingham, United Kingdom ^6^Hereford and Worcestershire Health and Care NHS Trust, Worcester, United Kingdom ^7^Division of Psychology and Mental Health, University of Manchester, Manchester, United Kingdom ^8^Mersey Care NHS Foundation Trust, Merseyside, United Kingdom ^9^Early Intervention Service Flyde and North, Lancashire and South Cumbria NHS Foundation Trust, Preston, United Kingdom ^10^Institute of Mental Health, Division of Mental Health and Neurosciences University of Nottingham, Nottingham, United Kingdom ^11^Nottinghamshire Healthcare National Health Service Foundation Trust, Nottingham, United Kingdom ^12^Division of Health Sciences, Warwick Medical School, University of Warwick, Warwick, United Kingdom ^13^Institute of Clinical Sciences. College of Medical and Dental Sciences, University of Birmingham, Birmingham, United Kingdom ^14^Centre for Neuropsychiatric Genetics and Genomics, Division of Psychological Medicine and Clinical Neurosciences, Cardiff University, Cardiff, Wales, United Kingdom ^15^Division of Psychiatry, Imperial College London, London, United Kingdom ^16^Department of Health and Wellbeing, University of Warwick, Warwick, United Kingdom RU is Chief Investigator; and conceived the study, led the proposal and protocol development. TREB, MB, JTW, NH, NM and BCTU contributed to grant funding and study design. RD joined as co-lead in 2023. RD, EP, TW, RO, BW, SP and RU drafted the manuscript. All authors approved the final manuscript.
Name and contact information for the trial sponsor {5b}	Research Support Group – Research Governance Block B – Room 106 Aston Webb Building Edgbaston Birmingham B15 2TT http://www.researchgovernance@contacts.bham.ac.uk
Role of sponsor {5c}	The sponsor provided peer review of funding application. The sponsor has no role in the management, analysis, or interpretation of the data; writing of this report; and the decision to submit the report for publication.

## Introduction

### Background and rationale {6a}

#### Psychosis and depression

Psychotic disorders, including schizophrenia, can be highly disabling. They usually emerge in adolescence or early adulthood and are characterised by the onset of positive symptoms, including hallucinations, delusions and disordered thinking, and negative symptoms, such as poor motivation. There are over 12,000 new cases of first-episode psychosis (FEP) in England annually, with significant increases seen since COVID-19 [1]. The burden on the individual, their family and society is large, with a cost to the UK economy of more than £11 billion per year [2, 3]. Beyond the impact of the positive and negative symptoms of FEP, around 40% of people experience a moderate or severe depressive episode following FEP [4, 5]. Studies show that positive symptoms respond to current treatment for many, but despite a reduction in symptoms, a decline in functioning, usually attributed to the persistence of negative symptoms, is still common. This suggests that current treatment regimens are limited in their ability to improve outcomes. Our hypothesis is that depression, in addition to negative symptoms, in the early stages of psychosis, contributes to functional decline and is a key area of unmet need [6, 7].

Considerable evidence suggests that depression in FEP has long-term adverse consequences for social and occupational recovery, quality of life, and risk of relapse [6, 8, 9]. Follow-up studies have found that fewer than 40% of patients with FEP achieve full recovery, and up to 70% remain out of employment, education, and training, even with existing, intensive interventions [9, 10]. A number of longitudinal studies of FEP [11–17], with a mean follow-up of 3 years, demonstrated a clear and significant association between depression and both poor functional outcomes and reduced quality of life following FEP. Additionally, depression after FEP has a long-term impact on the likelihood of suicidal behaviour, with the effect lasting up to 7 years [18]. Depression is the most significant risk factor for suicidal behaviour and completed suicide in FEP, with over a third of patients attempting suicide and completed suicide being most common in the earlier stages of the illness [19, 20].

Furthermore, a number of studies have found that depression and anxiety often precede a psychotic relapse, suggesting that these ‘co-morbidities’ may precipitate the development of positive symptoms such as hallucinations and delusions [21]. One possible implication is that targeted treatment for depression could play a role in preventing psychotic relapse. For example, symptoms of depression, such as poor motivation and social withdrawal, might be expected to reduce engagement with focused interventions addressing recovery and relapse prevention after FEP.

Thus, treatment targeted at the prevention of depression after FEP could be an important strategy, with a beneficial impact on functional recovery, risk of suicide and quality of life, as well as secondary benefits related to the prevention of psychotic relapse. Given the potential positive impact on patients, carers, families, the NHS, and society, this prevention strategy, if successful, is likely to be a cost-effective intervention.

#### Past research

Recent observational studies have evidenced the frequency and importance of depression following FEP [9]. There is substantial meta-analytic evidence that antidepressants are well-tolerated when prescribed in combination with continuing antipsychotic medication; Helfer et al. [22] reviewed the co-prescription of antidepressants with antipsychotic medications, for any clinical indication, in 3068 participants with schizophrenia and showed an overall beneficial effect with a low risk of adverse effects [22]. A 2017 systematic review [23] identified 15 studies that reported the effect of antidepressant medication specifically for the treatment of a depressive episode in schizophrenia; eight studies investigated selective serotonin reuptake inhibitors (SSRIs), with the majority of these showing effectiveness and an overall number needed to treat (NNT) of 5 [23]. In addition, in moderate or severe depressive disorders without psychosis, a recent large-scale meta-analysis has demonstrated the clear effectiveness of antidepressant medication, with sertraline, agomelatine, amitriptyline, and escitalopram being the medications considered to be the most effective and best tolerated [24].

Regarding other treatments for depression in psychosis, cognitive behavioural therapy for psychosis (CBTp) has been the subject of much research in recent years [25]. However, this body of evidence has primarily focused on its effectiveness for positive symptoms or the prevention of transition from high-risk status to psychosis. The secondary evidence for the effect of CBTp on depression in psychosis is mixed, with a small effect size at best [9, 26]. Prevention of depression using antidepressant medication is an established strategy in other branches of medicine for groups at high risk of depression, with low-dose antidepressant medication used to reduce the incidence of depression post-stroke [27], after a cardiovascular event [28, 29], with liver disease [30], in post-partum women [31] and after traumatic brain injury [32]. SSRIs are the most frequently studied medication in these groups, with a reported reduction in the incidence of depression ranging from 20 to 50%.

In summary, the evidence base upon which clinicians and patients can make a choice about additional treatments in FEP remains limited. Whilst some evidence exists for the use of antidepressants to treat depression in people with schizophrenia, as well as those with other illnesses at equally high risk of developing depression, no previous study has specifically focused on preventing depression after FEP, despite this being a period with very high rates of depression associated with poor outcomes relating to suicidal behaviour, relapse, quality of life, and poor functional recovery.

## Objectives {7}

### Research hypothesis

The use of an SSRI antidepressant alongside usual antipsychotic medication will be an effective and cost-effect intervention for preventing depression post-FEP. The rationale is that preventative treatment will reduce the risk of an individual developing depression and thus prevent additional morbidity, ultimately improving recovery, function, and quality of life and potentially reducing resource use.

### Primary objective

This study aims to assess the clinical of an SSRI antidepressant medication (sertraline) for the prevention of a depressive episode following FEP.

### Secondary objectives

This study aims to assess the effectiveness of an SSRI antidepressant medication (sertraline) for other important outcomes, including anxiety, positive and negative symptoms of psychosis, functional recovery, and quality of life following FEP, as well as the cost-effectiveness of the intervention.

### Trial design {8}

The trial design is as follows: multi-centre, 1:1 randomised, double-blind, placebo-controlled trial of sertraline 50 mg once a day for 6 months investigating its efficacy for the prevention of depression. All participants and their research teams will be unblinded following the 6-month outcome assessment or, if they develop depression, the primary endpoint, prior to this. Those participants recruited within the first 24 months of recruitment will undergo an additional 12-month follow-up visit (unblinded and observational). We include an internal pilot with clear stop–go criteria focusing on recruitment, completeness of assessment data and retention.

## Methods: participants, interventions, and outcomes

### Study setting {9}

The trial will take place in Early Intervention in Psychosis services (EIP) which are community-based, multidisciplinary teams that manage the vast majority of patients with FEP in England and Wales. Patients with FEP are required to be assessed and taken on by EIP within 2 weeks of presentation (NICE, 2015) [33].

### Eligibility criteria {10}

For each potential participant, eligibility for the trial will be confirmed by a medically qualified practitioner.

Inclusion criteria

- Diagnosis of first-episode psychosis (FEP)
- Within 12 months of initial treatment for FEP (as defined by the onset of care provision by an Early Intervention Team
- Positive and Negative Syndrome Scale [34]. (PANSS): individual positive symptom item scores all ≤ 4
- Sufficiently recovered from acute psychotic episode with the capacity to consent
- Males and females aged 18–65 years
- Currently prescribed antipsychotic medication at a stable dose
- Female participants must be willing to use one form of highly effective contraception

Exclusion criteria

- Current moderate or severe depression as indicated by a Calgary Depression for Schizophrenia Scale [35]. (CDSS) score > 7
- Currently prescribed antidepressant medication (or within 2 weeks of stopping if previously prescribed a monoamine oxidase inhibitor)
- Previous history of mania
- Contraindications to SSRI antidepressant treatment, e.g. recurrent thrombotic illness, previous adverse reaction, confirmed pregnancy, or planning to become pregnant (although the risk in pregnancy is low), prescribed pimozide
- (See sertraline summary of product characteristics (SmPC))
- Serious medical or neurological illness (as identified by a medically qualified doctor)
- Hypersensitivity to the active substance or any of the excipients or placebo
- Concomitant treatment with irreversible monoamine oxidase inhibitors (MAOIs)
- Patient with any systemic dysfunction (e.g. gastrointestinal, renal, respiratory, cardiovascular, neurological or psychiatric) or significant disorder which, in the opinion of the investigator, would jeopardise the safety of the patient by taking part in the trial
- Electrocardiogram (ECG): QTc interval > 450 ms recorded in the last 12 months

### Who will take informed consent? {26a}

It will be the responsibility of the Principal Investigator (PI) or delegate at each trial site to obtain informed consent for each participant prior to performing any trial-related procedure. Any delegation of this duty will be captured on the Site Delegation Log. PIs or delegate(s) will ensure that they adequately explain to the participant the study aims, the trial intervention, and the anticipated benefits and potential hazards of taking part in the trial. There is a two-stage information provision and consent process: patients will be given a copy of the Screening Patient Information Sheet (PIS) and sufficient time to consider the trial and discuss it with friends and family. If the participant expresses an interest in participating in the trial, they will be asked to return for a screening visit, where they will be asked to sign and date the latest version of the Screening Informed Consent Form (ICF) before any trial screening assessments are carried out. If eligibility is confirmed, the same process is then repeated with the main trial PIS and ICF.

### Additional consent provisions for collection and use of participant data and biological specimens {26b}

We will also request optional consent to allow linkage to patient data available in NHS routine clinical datasets, including primary care data (e.g. Clinical Practice Research Datalink, The Health Improvement Network, QResearch) and secondary care data (Hospital Episode Statistics) through NHS Digital and other central UK NHS bodies. The participant will consent to the Trial Office sending their name, address, date of birth, and NHS number to the relevant national registry and then for the national registry to link this to their data and send the information back to the Trial Office. The consent will also allow access to other new central UK NHS databases that will appear in the future. This will allow us (subject to receipt of additional funding via another grant application) to assess longer-term impact and health service usage data without needing further contact with the trial participants. Blood samples (9 ml) will be taken at 4 weeks for analysis to check concordance with trial medication. These blood samples will not be analysed until the trial intervention is complete for all participants to remove the risk of unblinding. If the participant consents to it, additional research blood samples (18 ml) will be taken and stored at the same time to be included in later genomic and biomarker studies.

## Interventions

### Explanation for the choice of comparators {6b}

The SSRI sertraline 50 mg daily is a commonly used antidepressant and the standard dose for men and women aged 18 years or older. Sertraline has clear evidence of efficacy in the treatment of moderate depression [24] and in the prevention of depression in other conditions [27]. It has a better safety profile in combination with antipsychotic medication compared with other effective SSRIs such as citalopram [36]. There is some evidence that women with chronic depression respond more favourably than men to sertraline [37]. FEP occurs more often in men (around 1.4 times higher) [38], and therefore, in our analytic plan, our stratification procedures will adjust for sex.

### Intervention description {11a}

Participants will take over-encapsulated tablets containing either 50 mg sertraline or a matching placebo. Sertraline is an SSRI, and 50 mg daily is the standard dose for men and women who are 18 years of age or older. The IMP manufacturer, Eramol, will source and re-encapsulate the sertraline 50 mg and manufacture the matched placebo, package, label, and distribute it to sites. A prescription will be provided to the site research pharmacy, and medication will be dispensed to the participant at the baseline visit and monthly thereafter for a further 5 months.

## Assessment schedule

### Screening assessment

Following consent for screening, participants will complete the screening assessments, including the CDSS, full PANSS, and a urine pregnancy test if they are female and of childbearing potential (fertile, following menarche and until becoming post-menopausal, unless permanently sterile).

### Baseline assessment

Following confirmation of eligibility, participants will formally consent to participate in the trial. Each participant’s contact details, medical history, current medications, demographic information, and vital signs (pulse, blood pressure and temperature) will be recorded. The full baseline battery of outcome measures will also be completed. Following these investigations and assessments, the participants will then be randomised.

### Follow-up visits

All follow-up visits will be conducted either at home or in outpatient clinics, tied in with routine appointments as much as possible. Participants will be followed up once every month for 6 months (and once at 12 months from randomisation if applicable).

#### One month

The participants will be asked questions about their medication adherence. The full battery of assessments that were completed at screening and baseline will be repeated. The *Mini International Neuropsychiatr﻿ic Interview* [39] *for DSM-IV (MINI)* will be completed if the CDSS score is > 5. If possible, a blood sample will be taken to monitor adherence and routine blood testing in the treatment of FEP, including LFTs, if these have not been completed in the last month as part of routine care. Vital signs (pulse, blood pressure and temperature) will also be recorded during this visit.

#### Two to 5 months

The CDSS (with MINI if CDSS score is > 5), PANSS positive symptom subscale, and the Generalised Anxiety Disorder Assessment [40] (GAD-7) will be completed.

#### Six months

The full battery of assessments that were completed at screening and baseline will be repeated. The MINI will be completed if the CDSS score is > 5. Data on health service usage and medication adherence will be collected from participants and from health records. The intervention will be unblinded to allow each participant and their care team to decide whether to continue or start sertraline. Vital signs (pulse, blood pressure and temperature) will also be recorded.

#### Twelve months

Participants recruited within the first 24 months of the recruitment period will be seen for one further follow-up at 12 months from randomisation (for participants recruited after 24 months of the recruitment period, their 6-month assessment will be the end of the trial). The full battery of assessments that were completed at screening and baseline will be repeated. The MINI will be completed if the CDSS score is > 5. Data on health service usage will be collected from participants and health records. The purpose is to chart the sustainability of any clinical benefit from sertraline.

Consent will include case record access, GP contact, and NHS Digital for future assessment of long-term impact.

### Criteria for discontinuing or modifying allocated interventions {11b}

A participant can withdraw from the study at any time without being required to provide the reasons. The participant will be given an opportunity to discuss the reason for withdrawal and any adverse events (AE). Any potential AE will be followed up by the principal investigator.

If an episode of depression is identified as defined by a CDSS score > 5 and confirmed by MINI, this will be recorded as an event, the intervention will be discontinued, and the participant will be unblinded to allow the appropriate treatment. The participant will remain in the trial, with their consent, for further assessments as per protocol.

Procedures for unblinding will follow Birmingham Clinical Trials Unit (BCTU) standard operating procedures. Participants will be unblinded if a reported event indicates that either treatment withdrawal or prescription of antidepressant medication is necessary.

### Withdrawal criteria

- The patient withdraws consent for any or no reason
- Any adverse event considered to be related to active trial medication which is a threat to health or well-being as determined by the local PI or the patient
- For safety reasons, as judged by the investigator
- The patient is unable to comply with the restrictions on the use of concomitant medications as listed on the sertraline summary of product characteristics (SmPC)
- The patient is unable to tolerate the study medication
- Participants who withdraw from active intervention will be offered the opportunity to continue with follow-up measures or withdraw completely from the study

### Strategies to improve adherence to interventions {11c}

Adherence to trial medication will be assessed using three methods: self-report at weeks 4 and 24, blood levels of sertraline at 4 weeks, and monitoring of prescription usage (pill counting, pharmacy dispensing records, returned medication).

### Relevant concomitant care permitted or prohibited during the trial {11d}

Participants may not be prescribed any other antidepressant medication during the trial, but concomitant care will be ‘treatment as usual’ for patients with first-episode psychosis. If a patient develops depression during the trial, this would be the primary endpoint reached, and the participant and their clinical team would be unblinded to allow care as usual. Concomitant treatment with irreversible monoamine oxidase inhibitors (MAOIs) is specifically contraindicated due to the risk of serotonin syndrome. Concomitant use of Sertraline in patients taking pimozide is contra-indicated. Any participants that become pregnant between the start of protocol-defined treatment and 30 days after the last dose will be recorded on a ‘notification of pregnancy’ form and followed up to the end of the pregnancy as per the applicable BCTU standard operating procedures. The participant’s GP would be notified that the participant is pregnant and taking part in the ADEPP trial and, therefore, potentially taking sertraline. However, there are no special interventions for patients taking sertraline in pregnancy.

### Provisions for post-trial care {30}

Unblinding will occur after the primary outcome assessment at 6 months or if a depressive episode requiring active treatment occurs prior to this. Following discussion with the clinical team, the participant will then continue with their usual care in EIP services.

## Outcomes {12}

### Primary outcome

The primary outcome will be the number of participants who have a depressive episode over the 6-month intervention phase. A depressive episode is defined as a new CDSS score of greater than 5, which is then confirmed by a MINI diagnostic interview. The proportion of participants who have a depressive episode over the 6-month period will be compared between the treatment and placebo arms.

### Secondary outcomes

We will measure a number of secondary outcomes:

- Suicidal behaviour is assessed via the Suicidal Behaviours Questionnaire-Revised [41] (SBQ-R), a 4-item validated tool that addresses the presence of suicidal ideation and attempts across the lifetime and preceding 12 months, together with current suicidal ideation and beliefs about future risk. Answers to the 4 questions gives a total score of 3–18. It has established linear total and cut-off scores to identify those with and without the reported risk. We will compare mean scores at 6 and 12 months between groups, adjusting for baseline value.
- Self-rated mood is assessed via the Quick Inventory of Depression Scale (Self Rating) [42] (QIDS-SR), a widely used, brief, general measure of depression. It gives a score between 0 and 27 and then an indicator of depression severity ranging from “indicate no depression” to incident very severe depression”. We will compare mean scores at 6 and 12 months between groups, adjusting for baseline value. It will be rated alongside the CDSS to allow comparison in secondary analysis with depression prevention trials in other disorders.
- The number of participants who have a depressive episode, as indicated by a CDSS score > 5 and confirmed by a MINI diagnostic interview at 12 months. The proportion of participants with a depressive episode will be compared.
- Anxiety will be assessed via the Generalised Anxiety Disorder Assessment [40] (GAD-7), a brief 7-item anxiety scale. It gives a score between 0 and 21, with cut-offs marking mild, moderate, and severe anxiety. We will compare mean scores at 6 and 12 months between groups, adjusting for baseline value.
- Anxiety will also be assessed via the State-Trait Anxiety Inventory [43] (STAI), a commonly used 20-item self-report scale for the assessment of trait and current (state) anxiety. It gives a score between 20 and 80. We will compare mean scores at 6 and 12 months between groups adjusting for baseline.
- The severity of positive symptoms is assessed via the Positive and Negative Syndrome Scale [34] (PANSS), an established 30-item, semi-structured interview for the assessment of the presence and change in symptoms of psychosis. We will compare mean scores of the positive symptom subsections of the PANSS at 6 and 12 months between groups adjusting for baseline.
- Improved functional outcomes are assessed via the Functional Remission of General Schizophrenia [44] (FROGS) and Social and Occupational Functioning Assessment Scale [45] (SOFAS). We will compare mean scores at 6 and 12 months between groups adjusting for baseline.
- Quality of life is assessed via the EQ-5D-5L [46], a 5-item health related quality of life assessment scale with well-established reliability and population norms [47] and the ICECAP-A [48], a 5-item measure of capability wellbeing. We will compare mean scores at 6 and 12 months between groups adjusting for baseline, as well as being used to supplement economic evaluation where the benefits of healthcare interventions may relate to the patient’s well-being more broadly defined.
- Rates of relapse of psychosis as defined by the number of people who have either a hospital admission or acute community care provided by a Home Treatment/Crisis Intervention team. Rates between the groups will be compared at 6 and 12 months.
- Side effects assessed via Antipsychotic Non-Neurological Side Effects Rating Scale-extended [49] (ANNSERS-e), the Barnes Akathisia Rating Scale [50] (BARS), and the Simpson-Angus Scale [51] (SAS) for drug-induced parkinsonism. We will compare mean scores at 6 and 12 months between groups adjusting for baseline.
- The resource use associated with prescribing antidepressant medication after FEP will be assessed using a modified version of the (Client Service Receipt Inventory—CSRI). The Client Service Receipt Inventory (CSRI) is a tool used to collect information on the whole range of services and supports study participants may use, including estimating the costs of an intervention [52].

Further exploratory analyses (e.g. by secondary outcome severity using established cut-off scores) and demographic (e.g. deprivation, ethnicity, sex) will be completed as data allows. All outcomes will be measured over the course of the intervention, at the end of the intervention (6 months), and at 6 months after the end of the intervention (12 months), where possible, to assess whether any beneficial effects are maintained. For the timing of all the various assessments, please see Fig. 2.

### Participant timeline {13}

**Fig. 1:**
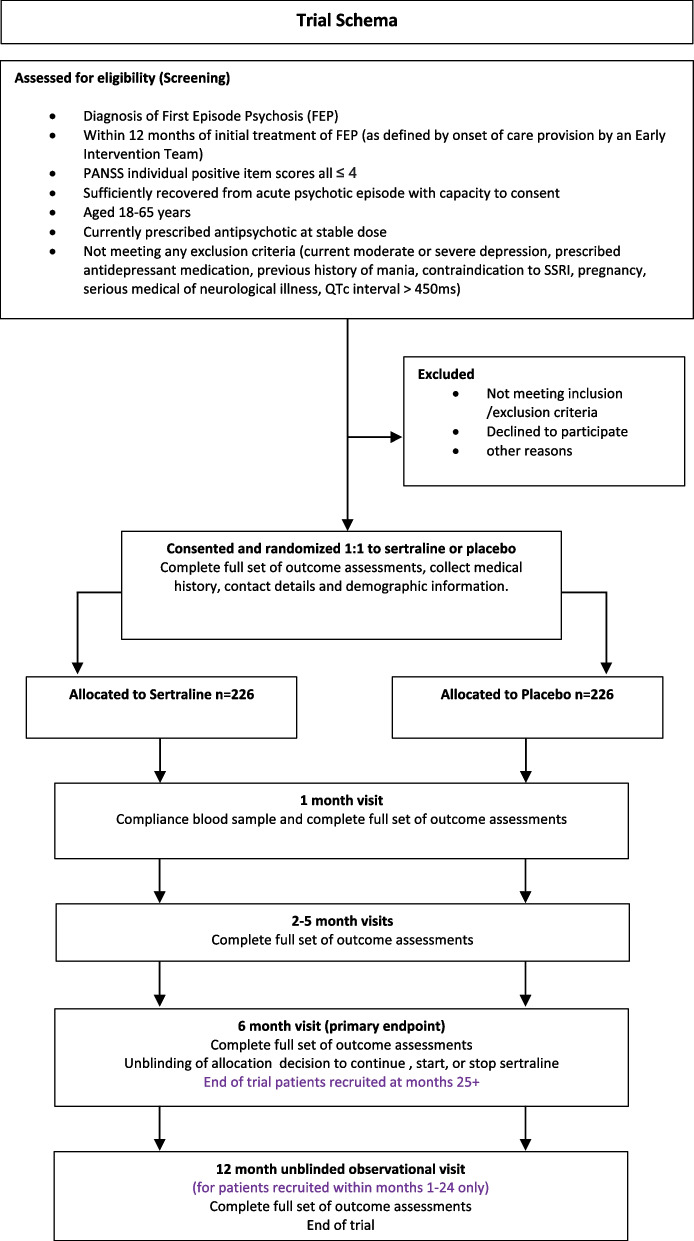
Trial scheme

See the trial scheme (Fig. 1).

### Sample size {14}

The sample size was calculated to look at a reduction from 40 to 25% in the number of participants who have a depressive episode over 6 months. This represents a relative reduction in depressive episodes of just over 35%, which, although relatively large, is average in the risk reductions seen in previous depression prevention trials (20–50%) [27–32]. A sample of 452 patients provides 90% to detect a reduction in the rate of depression from 40 to 25% at the 5% level whilst allowing for a 10% loss to follow-up at 6 months. Cumulative data suggests that attrition is less than 10%, so this is a conservative estimate [53]. Sample size calculations were performed using the Sample Size Tables for Clinical Trials Software, v1.0, and independently verified using the proc power statement in SAS v9.4.

### Recruitment {15}

We aim to recruit 452 participants from EIP services in England and Wales. In addition to the larger hubs, we will identify a number of smaller sites; this will make our recruitment more feasible and the results more generalisable. EIP team referrals are highly monitored by NHS England as part of the Access and Waiting Time Standards; thus, our initial inclusion criteria (diagnosis of FEP and within 12 months of treatment onset) will be straightforward to pre-screen from EIP team caseload information. Potential participants will be approached and informed of the trial by telephone call or at a routine clinical visit. They will be informed that their participation is voluntary and choosing not to participate will not affect their care.

## Assignment of interventions: allocation

### Sequence generation {16a}

Participants will be individually randomised on a 1:1 basis between sertraline and placebo via a secure online randomisation system based at Birmingham Clinical Trial Unit (BCTU). Randomisation will be provided by a secure online randomisation system at Birmingham Clinical Trials Unit (BCTU) (available at https://trials.bham.ac.uk/adepp). Randomisation will be minimised to ensure equal distribution of the most commonly prescribed antipsychotic medication in this population (olanzapine, risperidone, aripiprazole, and quetiapine) and sex. Randomisation Notepads will be provided to investigators to collate the necessary information for minimisation prior to randomisation. All questions and data items on the Randomisation Notepad must be answered before randomisation can be completed and a Trial Number is given. If data items are missing, randomisation will be suspended but can be resumed once the information is available. A ‘random element’ will be included in the randomisation algorithm to help reduce predictability and ensure concealment. This is a method used in the algorithm whereby each participant has a probability (unspecified here) of receiving the opposite treatment to the one they would have otherwise received under minimisation.

### Concealment mechanism {16b}

Allocation concealment will be ensured via remote allocation of trial codes. The randomisation process will be automated via the bespoke online database. The trial medication will be blinded (encapsulated sertraline 50 mg or placebo to match) from the clinical team members, participants/care providers, and researchers. Unique log-in usernames and passwords will be provided to those who wish to use the online system and who have been delegated the role of randomising participants as detailed on the Site Signature and Delegation Log. These unique log-in details will not be shared with other staff.

### Implementation {16c}

After participant eligibility has been confirmed and informed consent has been received, the participant can be randomised into the trial. At each randomisation, the site research team will access a secure online randomisation system to reveal a treatment pack number to match the participant’s randomised treatment. The randomisation sequence will be computer generated at the trial unit.

## Assignment of interventions: blinding

### Who will be blinded {17a}

This is a double-blinded trial, so the medication will be blinded from the clinical team members, participants/care providers, researchers, and data analysts. For data analysts, allocations will only be provided as groups “A” and “B” without reference to either intervention or placebo to enable the statisticians to remain blinded to allocation for interim and final analyses.

### Procedure for unblinding if needed {17b}

Participants and their clinical teams will be routinely unblinded at the primary endpoint (at 6 months) or additionally if (a) a depressive episode is identified (primary objective) before the primary endpoint, and (b) an event indicates that either treatment withdrawal or prescription of antidepressant medication is necessary.

At the primary endpoint (at 6 months), investigators with the unblinding role on the delegation log will be able to unblind participants at their site using the ADEPP database.

If emergency unblinding is required, this can be done by all investigators involved in patient care using the ADEPP database, which is available 24/7. All investigators responsible for patient care will be delegated the duty of conducting emergency unblinding on the delegation log. The reason for unblinding and the person requesting this will be recorded on the database, and an email will be sent confirming that unblinding has occurred.

## Data collection and management

### Plans for assessment and collection of outcomes {18a}

**Fig. 2:**
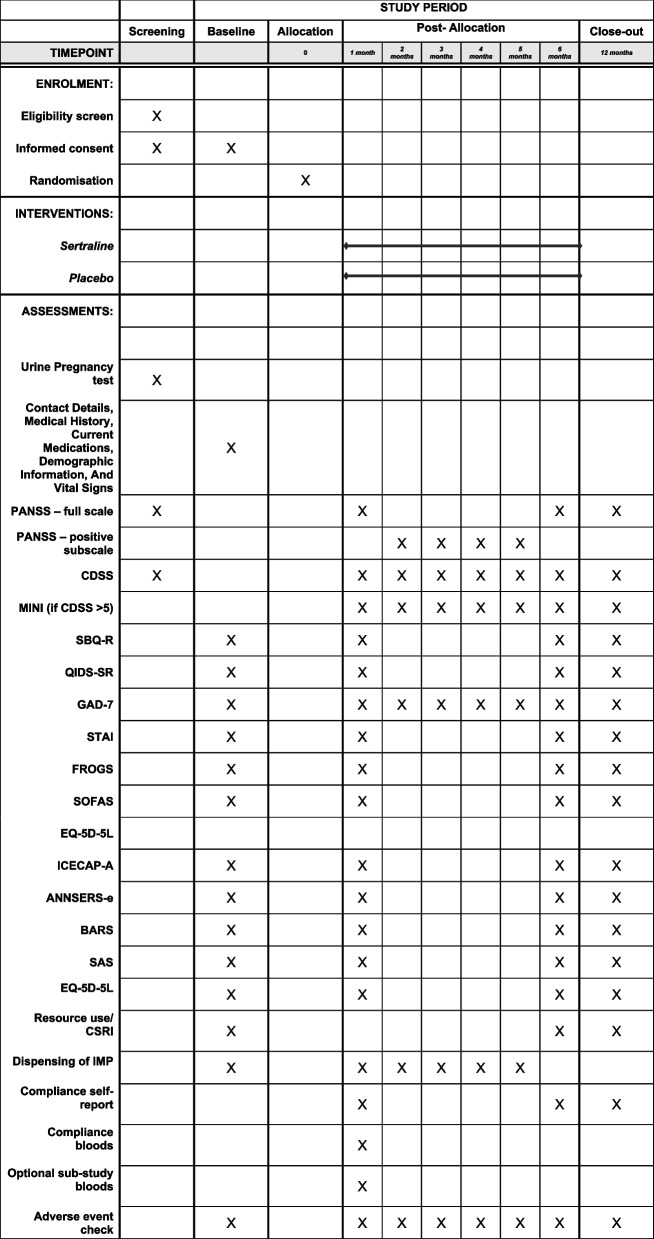
Schedule of enrolment, interventions, and assessments

All research investigators and staff are required to undergo extensive and documented training on the study protocol. Assessors of outcome measures will undergo specific training and assessment to ensure accuracy and standardisation. Study team members involved with the collection and/or shipment of blood will follow the study standard operating procedures (SOPs) for these processes. A description of each study instrument can be found in the outcomes section, and for information on when each assessment is conducted, please see Fig. 2.

### Plans to promote participant retention and complete follow-up {18b}

In our experience of longer and more complex trials [52, 54], we can expect a retention rate of 90% or higher, which has been accounted for in our sample size calculations. We are implementing several strategies to enhance retention and minimise loss to follow-up, including being flexible around time and location when scheduling appointments for follow-up and being responsive to any questions or concerns raised by the participants or carers. Those who withdraw from active intervention will be offered the opportunity to continue with follow-up measures or withdraw completely from the study.

### Data management {19}

Processes will be employed to facilitate the accuracy of the data included in the final report. These processes will be detailed in the trial-specific Data Management Plan (see Supplementary Materials). Coding and validation will be agreed upon between the trial manager, statistician, and programmer, and the trial database will be signed off once the implementation of these has been assured.

Data entry will be completed by the Trial Office via a bespoke BCTU trial database. The data capture system will conduct automatic range checks for specific data values to ensure high levels of data quality. Queries will be raised using data clarification forms (DCFs) via the trial database, with the expectation that these queries will be completed by the site within 30 days of receipt. Overdue data entry and data queries will be requested monthly.

ADEPP will use paper CRFs with data entry by the Trial Office.

### Confidentiality {27}

Personal data recorded on all documents will be regarded as strictly confidential and will be handled and stored in accordance with the Data Protection Act 2018.

Participants will always be identified using their unique trial identification number on the case report form and in correspondence between sites and BCTU. Participants will give their explicit consent for the secure movement of their consent form, giving permission for BCTU to be sent a copy. This will be used to perform in-house monitoring of the consent process.

The PI must maintain documents not for submission to BCTU (e.g. Participant Recruitment and Identification Log) in strict confidence. In the case of specific issues and/or queries from the regulatory authorities, it will be necessary to have access to the complete trial records, provided that participant confidentiality is protected.

BCTU will maintain the confidentiality of all participant’s data and will not disclose information by which participants may be identified to any third party. Representatives of the Trial Office and sponsor may be required to have access to participants’ notes for quality assurance purposes, but participants should be reassured that their confidentiality will be respected at all times.

### Plans for collection, laboratory evaluation, and storage of biological specimens for genetic or molecular analysis in this trial/future use {33}

At 4 weeks, if a participant consents, additional research blood samples (1 × 9 ml EDTA tube and 1 × 9 ml BD tube) will be taken alongside concordance bloods and stored de-identified for future genomic analysis and biomarker studies.

The BD tube will be processed and frozen in a – 80 °C freezer within 48 h of being taken. The EDTA tube will be processed as peripheral blood mononuclear cells and then frozen in a − 80 °C or lower freezer within 48 h of being taken. They will then be stored at the Human Biomaterials Resource Centre at the University of Birmingham. Both samples will be stored until they are either transferred on dry ice to Cardiff University for DNA extraction and genomic analysis or stored for future studies, which will be funded and ethically approved separately from the ADEPP trial.

## Statistical methods

### Statistical methods for primary and secondary outcomes {20a}

The analysis will be on an intention-to-treat basis. The primary outcome analysis will be the proportion of patients in the sertraline arm who become depressed compared with that in the placebo arm. For binary comparisons, a log-binomial model will be used to compare the proportions of depression in each arm at 6 months with adjustment for minimisation variables (4 most commonly prescribed antipsychotic medications and sex). Estimates of treatment effects will be shown as adjusted relative risk with 95% confidence intervals.

For secondary outcome analysis, those variables on a binary scale will be analysed in a similar way to the primary outcome. Those on a continuous scale will be analysed using a linear regression model to compare outcomes between the arms adjusting for minimisation variables. Adjusted mean differences between groups and 95% confidence intervals will be presented.

### Interim analyses {21b}

Interim analyses of safety and efficacy for presentation to the DMC will take place during the study. The DMC will meet prior to trial commencement to agree on the manner and timing of such analyses, but this is likely to include the analysis of the primary and major secondary outcomes and full assessment of safety (SAEs) at least at annual intervals. Criteria for stopping or modifying the trial based on this information will be ratified by the DMC. Details of the agreed plan will be written into the SAP.

### Methods for additional analyses (e.g. subgroup analyses) {20b}

We plan a sensitivity analysis controlling for a variety of clinical and demographic features known to be associated with higher rates of depression, including any previous depressive episode, sex, the severity of positive symptoms, socioeconomic deprivation, and aspects of treatment as usual (TAU) delivered from EIP services whilst participants are in the study including vocational and psychological interventions.

### Economic evaluation

Economic analysis of using sertraline for patients with FEP will comprise a trial-based economic evaluation from an NHS perspective and a model-based economic evaluation of the long-term impact on society. The trial-based economic evaluation will be based on the primary outcome, the cost-per-case of depression avoided, and the cost per quality-adjusted life year (QALY) gained. QALYs will be estimated using EQ-5D as favoured by NICE for assessing cost-effectiveness [55]. Results will be expressed as incremental cost-effectiveness ratios and cost-effectiveness acceptability curves to represent the probability of being cost-effective at different willingness to pay thresholds. The model-based economic evaluation will estimate the long-term cost-effectiveness of sertraline to society, additionally examining impacts on patients’ wellbeing, estimated using the ICECAP-A capability measure [56], family carers, and resource use outside the healthcare setting.

### Methods in analysis to handle protocol non-adherence and any statistical methods to handle missing data {20c}

Every attempt will be made to collect complete follow-up data on all participants; it is thus anticipated that missing data will be minimal. For participants with missing primary outcome data, we plan to impute the CDSS score in the event the 6-month data is missing to ensure that everyone randomised is included in the analysis. Sensitivity analyses will be undertaken, including a complete case analysis and per protocol analysis. Further sensitivity analyses will explore the assumptions made around those missing data and include a ‘tipping-point’ analysis.

### Plans to give access to the full protocol, participant level-data and statistical code {31c}

Only the trial management group (TMG) will have access to the full trial dataset to ensure that the overall results are not disclosed by an individual trial site prior to the main publication. Following the publication of the findings, the final trial dataset will be made available to external researchers upon approval from the TMG and the BCTU data-sharing committee in line with standard data-sharing practices for clinical trial data sets.

## Oversight and monitoring

### Composition of the coordinating centre and trial steering committee {5d}

A trial steering committee (TSC) will be created for the ADEPP trial and meet remotely or in person, as required depending on the needs of the trial. Membership and duties/responsibilities are outlined in the TSC Charter (see Supplementary Materials). In summary, the TSC will provide overall oversight of the trial, including the practical aspects of the trial, as well as ensuring that the trial is run in a way which is both safe for the participants and provides appropriate data to the Sponsor and investigators.

The data monitoring committee (DMC) aims to protect and serve ADEPP patients with regard to safety, to assist and advise chief investigators so as to protect the validity and credibility of the trial, to safeguard the interests of trial participants, assess the safety and efficacy of the interventions during the trial, and monitor the overall conduct of the clinical trial. The DMC will receive and review information on the progress and accruing data of this trial and provide advice on the conduct of the trial to the trial steering committee (TSC). Membership and duties/responsibilities are outlined in the DMS Charter (see Supplementary Materials).

### Composition of the data monitoring committee, its role and reporting structure {21a}

Data analyses will be supplied in confidence to an independent data monitoring committee (DMC), which will be asked to give advice on whether the accumulated data from the trial, together with the results from other relevant research, justifies the continuing recruitment of further participants. The DMC will operate in accordance with a trial-specific charter. The DMC will meet at least annually as agreed by the committee and documented in the Charter. More frequent meetings may be required for a specific reason and will be recorded in minutes.

Additional meetings may be called if recruitment is much faster than anticipated, and the DMC may, at their discretion, request to meet more frequently or continue to meet following the completion of recruitment. An emergency meeting may also be convened if a safety issue is identified. The DMC may consider recommending the discontinuation of the trial if the recruitment rate or data quality are unacceptable or if any issues are identified that may compromise participant safety. The trial will stop early if the interim analyses reveal differences between treatments that are deemed to be convincing to the clinical community.

### Adverse event reporting and harms {22}

The collection and reporting of adverse events (AEs) will be in accordance with the UK Policy Framework for Health and Social Care (2017), the Principles of GCP as set out in the UK Statutory Instrument (2004/1031 and subsequent amendments), the requirements of the Health Research Authority (HRA), and the Medicines for Human Use (Clinical Trials) Regulations 2004 and amendments thereof. Adverse event (AE) checks will occur at every visit, and the reporting period for AEs in ADEPP will be from the day of consent until 30 days after the last dose of trial treatment. All medical occurrences which meet the definition of an AE during the reporting period should be reported on the AE log and returned to the Trial Office.

Some harms, such as potential side effects from medications, including nausea, agitation, suicidal thinking, and serotonin syndrome, will be collected systematically and have been embedded in the data collection process in the monthly visits by the use of validated scales such as BARS, ANNSERS-e, and SAS. Non-systematic collection includes the use of open questions where the participant is able to voice any concerns or changes they would like to report, and from information becoming known outside monthly contact, for example, via liaison with the clinical team and will be logged on participant records.

Serious adverse events (SAEs) are classified in one of three ways: as safety reporting exempt SAEs, which require the PI to record in medical notes but not report to BCTU (e.g. pre-planned hospitalisation), those that require recording in notes and reporting to the trial office in a non-expedited manner within 4 weeks of becoming aware of the event on a trial-specific SAE form (e.g. attendance at A&E for a mental health-related reason, referral to mental health crisis team, referral to liaison psychiatry), or any other events which required recording in notes and reporting to the trial office in an expedited manner, i.e. within 24 h of first awareness.

### Frequency and plans for auditing trial conduct {23}

The research sites are monitored by the sponsor in accordance with the trial risk assessment and monitoring plan. On-site monitoring visits by the sponsor may be triggered by several factors (e.g. poor CRF return, poor data quality, low or high SAE reporting rates, and an excessive number of participant withdrawals or deviations). Monitoring activities are reported to the coordinating centre and any issues noted and followed up to resolution. BCTU routinely check incoming ICFs and CRFs for compliance with the protocol, data consistency, and missing data. Sites are sent data queries requesting missing data or clarification of inconsistencies or discrepancies monthly.

### Plans for communicating important protocol amendments to relevant parties (e.g. trial participants, ethical committees) {25}

Any substantial amendments to the trial protocol will be submitted for review to the Research Ethics Committee (REC), Health Research Authority (HRA), Medicines and Healthcare Products Regulatory Agency (MHRA), and Research and Development Department at the trial sites. If a substantial amendment affects a participant’s decision to continue in the trial, this new information will be communicated to them by the local research team. If the participant is willing to continue, consent is regained.

### Dissemination plans {31a}

The investigators are responsible for publicly disseminating results, study materials, and procedure manuals.

The trial is registered at (ISRCTN12682719). The results of this trial will be disseminated through publication in a peer-reviewed journal, presentations, and through the study website https://adepp-study.digitrial.com/.

## Discussion

Adding sertraline to any standard treatment regimen for FEP has the potential to prevent the development of depression, a common co-morbidity, after the onset of FEP. By doing so, it is possible that there will be a reduction in the illness burden of FEP with longer-term benefits, including a reduction in the risk of anxiety, suicidal behaviour, and relapse. Thus, this medication strategy has the potential to produce a significant improvement in the level of functioning and quality of life for FEP patients, both within the first 12 months and beyond. If proven to be effective, this could be a very cost-effective strategy for improving outcomes for those with FEP in both the short and long-term, having a positive impact on many individuals as well as reducing the cost of care and other implications for society.

## Trial status

The trial is active, and recruitment is ongoing. Recruitment commenced in July 2021 and is expected to continue until June 2024. The trial is on protocol version V11.0 dated 07/12/2022.

### Supplementary Information

**Additional file 1.**

**Additional file 2.**

**Additional file 3.**

## Data Availability

Initially, only the members of the study group will have access to the final trial dataset whilst the results are analysed and published.

## Abbreviations

FEP: First-episode psychosis
ADEPP: Antidepressant for the prevention of Depression following first-episode psychosis
EIP: Early Intervention in Psychosis
CBTp: Cognitive behavioural therapy for psychosis
SSRIs: Selective serotonin reuptake inhibitors
TAU: Treatment as usual
PANSS: Positive and Negative Syndrome Scale
CDSS: Calgary Depression for Schizophrenia Scale
MAOIs: Monoamine oxidase inhibitors
AE: Adverse events
LFT: Liver function tests
BCTU: Birmingham Clinical Trials Unit
SBQ-R: Suicidal Behaviours Questionnaire-Revised
QIDS-SR: Quick Inventory of Depression Scale
GAD-7: Generalised Anxiety Disorder Assessment
STAI: State-Trait Anxiety Inventory
FROGS: Functional Remission of General Schizophrenia
SOFAS: Social and Occupational Functioning Assessment Scale
ICECAP-A: Investigating Choice Experiments CAPability measure for Adults
BARS: Barnes Akathisia Rating Scale
ANNSERS-e: Antipsychotic Non-Neurological Side Effects Rating Scale-extended
SAS: Simpson-Angus Scale
CSRI: Client Service Receipt Inventory
