# Supplementary material for: Antidepressants for the prevention of depression following first-episode psychosis (ADEPP): study protocol for a multi-centre, double-blind, randomised controlled trial
Source: Trials. 2023 Oct 6;24:646. doi: 10.1186/s13063-023-07499-3 (PMC10557320; doi:10.1186/s13063-023-07499-3)
Supplement: Supplementary file 1 — Additional file 1. [file 13063_2023_7499_MOESM1_ESM.docx]

| **Data Management Plan** | | | | |
| --- | --- | --- | --- | --- |
| **Trial Details** | | | | |
| **Name of Trial:** | | ADEPP | | |
| **Chief Investigator:** | | Prof. Rachel Upthegrove | | |
| **CRF completion** | | | | |
| Parameter | | | Description of plan | |
| Indicate whether trial is using paper or electronic CRFs | | | Paper CRF with online randomisation available for sites. | |
| Have the site been provided with guidance on how CRFs should be completed, who should complete them and how to make corrections? Provide version of CRF completion guidelines or detail of information provided? | | | Yes, the trial manager will address these points during the SIV with each site.  Case Report Form (CRF) completion is in accordance with the CRF Completion Guide BCTU-GDL3 (for site staff) and the Data Entry Work Guide v1.0 (for BCTU staff). | |
| Has the site delegation log been obtained? | | | Delegation logs will be obtained from all sites and staff completing CRFs will be checked against the logs received from each site.  Site staff will only be given access to the online randomisation application on receipt of delegation log with the relevant task assigned to them and signed off by the PI. | |
| **Source Documentation** | | | | |
| Parameter | | | Description of plan | |
| Have the source documents for the trial been identified? Please detail where this agreement has been made (trial protocol, Source Data agreement etc.) | | | Yes, detailed in the protocol, section 11.1. This will be discussed in site initiation visits and evidenced in their site initiation checklist. | |
| **CRF returns and tracking** | | | | |
| Parameter | | | Description of plan | |
| How will CRFs be returned to BCTU? | | | CRFs are completed at site and can be sent as either original or True Copy in the post, or by True Copy via email scan. | |
| At what frequency will reminders be sent where CRFs have not been returned? | | | Sites will be requested to compete CRFs when they are expected on the system via email. These reminders will include a report of outstanding CRFs per timepoint per TNO that are yet to be received by the trials unit.  These requests will be sent by the data manager monthly. | |
| What method will be used to track CRFs? | | | CRFs arriving via post should be date stamped on the day of arrival at the ADEPP trial office. CRFs arriving via email should be printed on the date of receipt and date stamped accordingly  The form return rates are visible via the database using the forms management feature, so poor performing sites or problematic CRFs, in terms of timely return, can be identified. These are reviewed and acted on periodically by the trial manager, and are reviewed regularly by the trial management group.  An overall centre return rate of <85% for a centre at a given time point will prompt corrective action (e.g. trial manager organising extra training with the site staff (research nurse and PI) and escalation to senior members of the team (senior trial manager, team leader and CI) where return rates do not improve.  If the return rate drops to <70%, or remains at <85% for the 3 months after the poor return rate is initially identified, the site will be visited unless a specific and correctible reason is given by the site (e.g. long term staff absence).  Data Clarification Forms (see below for process) can similarly arrive as both original and True Copy and staff are trained to process their receipt and entry in the same manner as for CRFs. | |
| **Data entry** | | | | |
| Parameter | | | Description of plan | |
| Indicate where data entry will be performed – at site or at BCTU | | | Data is entered by trained members of the central ADEPP trial team at BCTU who have been assigned the role and a log in, and specifically provided with access permission for the ADEPP database. | |
| Detail training that has been provided to those performing data entry | | | BCTU data managers have been given personal data entry training by the trial manager (this may also be delegated to an experienced data manager).  Data Managers are required to read and record that they have understood the TM-4 SOP on data management and are given trial specific training in relation to the disease, form and data contingencies, and checking the names of the person completing the forms against the delegation log for the relevant site. | |
| Detail any data entry testing that has been conducted | | | The ADEPP database is a bespoke database created by BCTU Programming Team in accordance with the relevant SOPs at the time and is tested by both the Programming Team and the Users prior to release. Documents relating to this testing are maintained by the Programming Team. | |
| Will the delegation log be checked to ensure that only individuals with the delegated duty have completed CRFs/DCFs? | | | Delegation logs will be obtained from all sites and staff completing CRFs will be checked against the logs received from each site. DCFs are typically sent to the individual at site who completed the CRF, and will always be sent to individuals with the relevant role on the site delegation log. | |
| **Data queries** | | | | |
| Parameter | | | Description of plan | |
| What is the data query process? Have the site been provided with guidance on how DCFs should be completed?  Include detail on how queries will be raised and by what method they will be notified e.g. data clarification form (BCTU-QCD36) | | | Data queries will be generated within the database itself. If a data field requires a query the individual field is selected and a query raised against it.. This also enables the recording of the original date (first chase date) the query was raised.  The query will be marked as active by the Data Manager and a log of all active queries are exported to an excel spreadsheet and sent as DCFs to each site monthly via email  For each query that has been addressed by the site, the Data Manager will change the data in the CRF on the database to marry the information provided by the site in response to the query and in turn this will change the status of the query to pending resolution. Lastly the data manager will close the query once resolved.  .  DCFs are returned to the ADEPP trial team either via email or post by members of the site staff who are on the delegation log and have been assigned the roles of CRF completion and correction.  The state of any data query can be identified at any point and reports can be generated to show timeliness of responses by site or by form.  Critical data item queries are considered resolved once the data has been provided or the site has clearly stated that the data is unavailable. For non-critical data items sites will be requested to correct any errors or omissions on initial processing of the CRF, and if no adequate response is forthcoming they will be requested two more times (up until the 3^rd^ chase). Data Manager will change the date each month when it is resent. If there is still no adequate response after the 3rd chase then the data in question will be categorised as missing and the query closed. | |
| At what frequency will data queries be sent to site? | | | DCFs are sent to sites in batches at the end of each month. With the exception of SAEs, which are processed immediately upon receipt, | |
| Who will be responsible for generating and forwarding the data queries to site? | | | The day-to-day management of the DCF process is the responsibility of the Data Manager. | |
| At what frequency will reminders on unanswered queries be sent? | | | Since the batching of DCFs is predicated on each form being either “open” or “closed”, sites will continue to receive reminders about outstanding approximately every 4 weeks until resolution. With the exception of non-critical data items that have been chased up three times without a response, at this point they will be closed and no longer chased. | |
| **Data Entry Quality Control Checks (DEQC) and Data Validation** | | | | |
| Parameter | | | Description of plan | |
| Describe the type, percentage and frequency of DEQC that will be made on the data. | | | DEQC on data entered by the BCTU ADEPP trial team can be performed by any members of the ADEPP Trial team but should preferentially be done by someone other than the team member making the original data entry. There may be specific times when this is not possible.  A minimum of 1% DEQC for each type of CRF (or 5 forms, whichever is greater) will be performed with checks being performed every 12 months and additionally when new data entry staff start in post, or significant amendments are made to CRFs.  The selection of this ≥1% will be chosen at random and checked. .A log of these checks will be maintained in a excel spreadsheet which will be updated with each annualDEQC check. PDFs will be produced to each annual check t and filed in the TMF. | |
| What validation checks will be used? | | | Data in ADEPP will be validated according to the Data Validation Plan at the time of data entry. In addition to the DEQC the ADEPP database has a number of built in validation rules, warnings and range parameters.Statistical validation checks will be performed periodically. | |
| Who will check the consistency of the data? | | | Please see above – data consistency will be checked as part of the DVP and statistical validation | |
| Outline acceptable error rates for primary and secondary outcomes and the process that will be implemented if error rates exceed acceptable levels | | | For ADEPP the acceptable error rate is 2%.  In the event that the error rate for any form exceeds 2%, then further checks will be made up to the 5%, then 10% and eventually 100% level, if the greater than 2% error rate persists. Note that whilst form numbers are low and where the number of data fields per form are also low, there may be times when just a single error will produce an unacceptable error rate. In this situation further data will be monitored until it is clear whether a problem exists.  In terms of escalation, if unacceptable error rates can be related to a particular user, then further training will be provided, whilst if the error rate appears only for particular forms, then consideration will be given to re-designing the CRF. | |
| **Self-evident corrections** | | | | |
| Parameter | | | Description of plan | |
| What type of self-evident corrections can be made? | | | Changes to administrative notes and reference numbers: when new information becomes available such that a reference number does not accurately reflect the sequence of CRFs received e.g. an SAE form is received for an incident which occurred prior to an already reported incident, then it is appropriate to change the reference number provided no DCFs have been raised using the original number.  Any notes relating to the patient care which have an impact on the administration process, but not the data fields themselves, can be changed as appropriate. | |
| Outline the self-evident corrections that have been agreed with site PI (BCTU-GDL5 – currently under development) | | | Self-evident corrections are detailed in the protocol. | |
| **Data storage and protection** | | | | |
| Parameter | | | Description of plan | |
| Indicate where your data is being stored? Outline how both electronic and paper trial documents are stored in accordance with the Data Protection Act 2018 | | | All information will be securely stored under the provisions of the provisions of the General Data Protection Regulation (GDPR) (EU) 2016/679 and/or applicable laws and regulations, and the NHS Code of Confidentiality will be observed.  The details of each participant will be entered onto a dedicated secure trial database, specifically constructed for the purpose. The BCTU is highly experienced in the storage and management of confidential data and utilises advanced security systems to protect personal details of research records. Trial data provided to the oversight Committee will be anonymised.  Online data transfer to the University of Birmingham will use SSL encryption (equivalent to internet banking). Individual investigators at participating centres will have their own username and password and will only have access to their own patients' data. Study Office staff will have access to all data via secure university network.  Access to the BCTU is restricted to authorised personnel by swipe card access. Individual rooms and cabinets that store trial information are locked when not in use. | |
| Detail any data backup system in place to guard against loss of data due to software or environmental disasters | | | Data is automatically backed up each night to the College of Medicine and Dentistry file share, and then onto tapes which are kept in a fire proof safe. | |
| Describe the processes that are in place to ensure that patient confidentiality is being maintained with trial patient data | | | The Trial Office will maintain the confidentiality of all patient data and will not disclose information by which patients may be identified to any third party other than those directly involved in the treatment of the patient and organisations for which the patient has given explicit consent for data transfer.  The patient consent form, which will be sent to the BCTU will, out of necessity, contain identifiable personal data. Patients will be informed of this and be asked to confirm their consent for this. The consent form will be sent (and stored) separately to any CRF.  Trial Office staff will have access to all data via secure university network. Data analysis will be undertaken by statisticians at BCTU and the University of Birmingham | |
| Describe the process for ensuring participant identifiers are removed from data bases once patient identifiable information is no longer required | | | - Psuedonymised IDs (patient study numbers and record IDs) are encrypted using a one-way hashing algorithm.   • Names, addresses, post codes (which can be replaced by deprivation score instead) are removed  • All dates (DOB, DOD, study form/event dates) are replaced with an integer number calculated as days from randomisation date.  • All text fields are reviewed. Any that contain dates, names or other identifiers have those identifiers redacted. If identifiers proliferate in a field, the entire field is removed.  • Rare conditions recorded may be reviewed by the trial statistician and redacted from the data set. | |
| **HSCIC and ONS Data (if applicable)** | | | | |
| Parameter | | | Description of plan | |
| How long will you keep this data for? | | | Personal identifiable information (PII) is stored on the ADEPP database for the purpose of long term follow up by HSCIC and ONS data. No data has yet been received from HSCIC or ONS; there are no formalised plans to do so yet. | |
| Where will this data be stored in accordance with the data agreement? | | | Not yet applicable. | |
| Who has permission to view this data? | | | Only BCTU staff with additional permissions can view the PII in question. | |
| Outline the frequency the data agreement needs to be renewed? Who will be responsible for renewing the data agreement? | | | Not yet applicable. | |
| **Database lock** | | | | |
| Parameter | | | Description of plan | |
| Describe the database lock procedure, when this will occur and who will be responsible for informing the trial programmer | | | The database will be locked by Programming once the statistics team are satisfied that the last patient last visit has occurred, all data entry and coding has been completed, and all data queries have been resolved. The trial manager will inform the trial programmer, copying in the trial statistician and chief investigator, after gaining their approval | |
| **Archiving** | | | | |
| Parameter | | | Description of plan | |
| Describe the processes and location, for archiving both electronic and paper trial documentation at the end of the trial and the planned retention period of archiving | | | Archiving will be authorised by the BCTU on behalf of the Sponsor following submission of the end of trial report.  PIs are responsible for the secure archiving of essential trial documents (for their site) as per their NHS Trust policy.  All essential documents will be archived for a minimum of 25 years after completion of trial.  Archiving, at the time of writing, is performed within UoB, but any external providers selected in the future will be expected to comply with the applicable regulatory requirements. Destruction of essential documents will require authorisation from the BCTU on behalf of the Sponsor. | |
| Outline the long term storage plans if frequent access to the documentation is required before archiving | | | Study data will be stored within the BCTU under controlled conditions for at least 3 years after closure | |
| **Signatures** | | | | |
| **Form completed by** | **Signature** | | | **Date** |
| Ben Watkins |  | | |  |
| **Chief Investigator (Name)** | **Signature** | | | **Date** |
| Rachel Upthegrove | *Email signature* | | | 14/09/2022 |
