# Supplementary material for: Antidepressants for the prevention of depression following first-episode psychosis (ADEPP): study protocol for a multi-centre, double-blind, randomised controlled trial
Source: Trials. 2023 Oct 6;24:646. doi: 10.1186/s13063-023-07499-3 (PMC10557320; doi:10.1186/s13063-023-07499-3)
Supplement: Supplementary file 3 — Additional file 3. [file 13063_2023_7499_MOESM3_ESM.pdf]

ADEPP

Antidepressant for the prevention of DEPression following first episode Psychosis trial

Data Monitoring Committee Charter

Version 2.0

(developed using on MRC Clinical Trials Unit template DMC Charter version 2.01, 13-Mar-2006;
from DAMOCLES DMC Charter template v1, Feb 2005)

**Authorised by:**				
Name:	Rebecca Woolley		Role:	Senior Statistician
Signature:	Signed via email		Date:	31/08/2022
**Prepared by**				
Name:	Ben Watkins		Role:	Senior Trial Manager
Signature:	B Watkins (Signed electronically due to remote working)		Date:	31/08/2022

CONTENT	CHARTER DETAILS
**1. Introduction**	
Trial name and numbers	**Title:** Antidepressant for the prevention of DEPression following first episode Psychosis trial<br>**Short Title:** ADEPP<br>**Chief Investigator**: Professor Rachel Upthegrove, University of Birmingham<br>**Grant awarded by**: National Institute for Health Research, Health Technology Assessment (ref: NIHR127700)<br>**Sponsor**: University of Birmingham<br>**ISRCTN**: to be provided when available<br>**EUDRACT**: 2020-002787-32
Objectives of trial, including interventions being investigated	To establish the effectiveness and cost effectiveness of an antidepressant medication (sertraline) for the prevention of a depressive episode following first episode psychosis (FEP)<br><br>**Feasibility study outcome:**<br>Assess acceptability of the trial 12 months into recruitment. The rate of recruitment, participant retention and adherence rate, the percentage of eligible patients that are recruited and percentage of first 50 participants with usable data will be assessed.<br><br>**Main study primary outcome:**<br>The number of new cases of depression as indicated by a Calgary Depression for Schizophrenia Scale (CDSS) score of >5 and confirmed by Mini International Neuropsychiatric Interview (MINI) in each treatment arm over the 6-month intervention phase.<br><br>**Main study secondary outcomes:**<br>• Positive and Negative Syndrome Scale (PANSS) is the established 30 item semi-structured interview for assessment of the presence and change in symptoms of psychosis. It has both total score and subscales for positive, negative and general symptoms.<br>• Suicidal Behaviours Questionnaire- Revised (SBQ-R) is a 4 item validated tool to assess the presence of suicidal ideation and attempts in lifetime ever and preceding 12 months, together with current suicidal ideation and beliefs about future risk. It has established linear total and cut off scores to identify those with and without the reported risk.<br>• State-Trait Anxiety Inventory (STAI) is commonly used 20 item self-report scale for the assessment of trait and current (state) anxiety.<br>• General Anxiety Disorder (GAD7) is a brief 7 item anxiety scale specifically assessing generalised anxiety.<br>• Relapse of psychosis: as defined by a hospital admission or acute community care provided by Home Treatment/ Crisis Intervention team.<br>• Functioning: Global Assessment of Function (GAF) is a quick and simple scale to assesses the overall impact of psychological disturbance on functioning with subscales of disability related and

CONTENT	CHARTER DETAILS
	symptom related impairment, Given the complexity factors that may impact on functioning in emerging psychosis, the Social and Occupational Functioning Scale (SOFAS) and the Functional Remission of General Schizophrenia (FROGS) will also be rated.<br>• Quality of life: EQ-5D and ICECAP-A. EQ-5D is a 5 item health related quality of life assessment scale with well established reliability and population norms and the Investigating Choice Experiments Capability Measure for Adults (ICECAP-A), a more detailed measure of heath individual capability, used to supplement economic evaluation as the benefits of health and social care are not confined only to patients themselves.<br>• Economic: Healthcare resource use will be collected at each follow-up assessment, when patients will be asked to recall visits to health professionals, medications and admissions. The information provided will be checked by searching their electronic patient records, and these data will be recorded on a standard case report form based on the Client Service Receipt Inventory. Resource use will be costed using national sources.
Outline of scope of charter	The purpose of this document is to describe the membership, terms of reference, roles, responsibilities, authority, decision-making and relationships of the Data Monitoring Committee (DMC) for the this trial, including the timing of meetings, methods of providing information to and from the DMC, frequency and format of meetings, statistical issues and relationships with other committees.
**2. Roles and responsibilities**	
A broad statement of the aims of the committee	The aims of this committee are:<br>• To protect and serve ADEPP patients, especially with regard to safety, and to assist and advise Chief Investigators so as to protect the validity and credibility of the trial.<br>• To safeguard the interests of trial participants, assess the safety and efficacy of the interventions during the trial, and monitor the overall conduct of the clinical trial.
Terms of reference	The DMC should receive and review information on the progress and accruing data of this trial and provide advice on the conduct of the trial to the Trial Steering Committee (TSC).<br><br>The DMC should inform the Chair of the TSC if, in their view:<br>• The results are likely to convince a broad range of clinicians, including those supporting the trial and the general clinical community, that, on balance, one trial arm is clearly indicated or contraindicated for all participants or a particular category of participants, and there was a reasonable expectation that this new evidence would materially influence patient management.<br>or<br>• It becomes evident that no clear outcome would be obtained.

CONTENT	CHARTER DETAILS
Specific roles of DMC	Interim review of the trial's progress including updated figures on recruitment, data quality, adherence to protocol treatment and follow-up, and main outcomes and safety data. Specifically, these roles include to, but are not limited to:<br>• At the end of the internal feasibility phase, review the safety and recruitment data and advise as to whether the study should continue or not.<br>• Monitor evidence for treatment differences in the main efficacy outcome measures.<br>• Monitor evidence for treatment harm (e.g. toxicity, serious adverse events and reactions, deaths).<br>• Assess the impact and relevance of external evidence.<br>• Decide whether to recommend that the trial continues to recruit participants or whether recruitment should be terminated either for everyone or for some treatment groups and/or some participant subgroups.<br>• Decide whether trial follow-up should be stopped earlier.<br>• Assess data quality, including completeness (and by so doing encourage collection of high quality data).<br>• Maintain confidentiality of all trial information that is not in the public domain.<br>• Monitor recruitment figures and losses to follow-up.<br>• Monitor compliance with the protocol by participants and investigators.<br>• Consider the ethical implications of any recommendations made by the DMC.<br>• Monitor planned sample size assumptions.<br>• Suggest additional data analyses if necessary.<br>• Advise on protocol modifications proposed by investigators or sponsors (e.g. to inclusion criteria, trial endpoints, or sample size).<br>• Monitor continuing appropriateness of patient information.<br>• Monitor compliance with previous DMC recommendations.
**3. Before or early in the trial**	
Whether the DMC will have input into the protocol	Before recruitment begins the trial will have undergone review by the funder/sponsor (e.g. peer review for public sector trials), and a research ethics committee (REC). Therefore, if a potential DMC member has major reservations about the trial (e.g. the protocol or the logistics) they should report these to the trials unit and may decide not to accept the invitation to join. DMC members should be independent[1] and constructively critical of the ongoing trial, but also supportive of aims and methods of the trial.
Whether the DMC will meet before the start of the trial	The DMC will first meet before the trial starts or early in the course of the trial, to discuss the protocol, the trial, the analysis plan, future meetings, and to have the opportunity to clarify any aspects with the

[1] Independence is defined in the table in Annexe 1

CONTENT	CHARTER DETAILS
	Chief Investigator (CI) and Trial Management Group (TMG). The DMC should meet within one year of recruitment commencing.
Any issues specific to the disease under study	There are no specific issues of the disease/illness under study.
Any specific regulatory issues	The DMC should be aware of any regulatory implications of their recommendations.
Any other issues specific to the treatment under study	There are no specific treatment issues.
Whether members of the DMC will have a contract	DMC members will not formally sign a contract but should formally register their assent to join the group by confirming (1) that they agree to be on the DMC and (2) that they agree with the contents of this charter. Any competing interests should be declared at the same time. Members should complete and return the form in Annexe 1.
**4. Composition**	
Membership and size of the DMC	The members of the DMC for this trial are:<br>(1) *Chair: Professor Stephen Lawrie (Professor of Psychiatry, University of Edinburgh)*<br>(2) *Professor Eileen Joyce (Professor of Neuropsychiatry, University College London)*<br>(3) *Ulrike Naumann (Consultant Strategy/Life Science, IQVIA)*<br>The members should be independent of the trial (should not be involved with the trial in any other way or have any competing interest(s) that could impact on the trial). Any competing interests, both real and potential, should be declared. A short competing interest form should be completed and returned by the DMC members to the trial coordinating centre (Annexe 1).
The Chair, how they are chosen and the Chair's role. (Likewise, if relevant, the vice-Chairman)	The Chair will have previous experience of serving on DMCs, experience of Chairing meetings and will be able to facilitate and summarise discussions. Dr Stephen Lawrie will chair, facilitate and summarise discussion of the DMC. A vice-Chair will not be appointed.
The responsibilities of the DMC statistician	The DMC membership will include a statistician to provide independent statistical expertise, especially with regards to interpretation of accumulating data and guidance through the report. The DMC statistician will not prepare the DMC report.
The responsibilities of the trial statistician	The trial statistician will have overall responsibility for the production of the report to the DMC and will participate in DMC meetings, guiding the DMC through the report, participating in DMC discussions and, on some occasions, taking notes.

CONTENT	CHARTER DETAILS
The responsibilities of the trials unit team	The trial statistician will produce the report to the DMC and will participate in DMC meetings, guiding the DMC through the report, participating in DMC discussions and, on some occasions, taking notes. The Trial Manager may attend open sessions of the meeting.
The responsibilities of the Chief Investigator (CI) and other members of the Trial Management Group (TMG)	The CI may be asked, and should be available, to attend open sessions of the DMC meeting. The other TMG members will not usually be expected to attend but can attend open sessions when necessary (see organisation of DMC meetings).
**5. Relationships**	
Relationships with Chief Investigators, other trial committees (e.g. Trial Steering Committee (TSC) or Executive Committee), sponsor and regulatory bodies	The responsibilities of each trial group are detailed in the protocol. The relationships between these groups are displayed in Figure 1.
Clarification of whether the DMC is advisory (make recommendations) or executive (make decisions)	It is customary that the DMC does not make decisions about the trial, but rather makes recommendations to an appropriate executive committee (e.g. the TSC and TMG) or its Chair.
Payments to DMC members	Members of the DMC will be reimbursed for reasonable expenses incurred when attending TSC meetings. Motoring expenses will be reimbursed at the rate in use by the Finance Department of the University of Birmingham at the time of the meeting. Fines or penalties will not be reimbursed. Rail travel will be reimbursed to the level of a standard class return ticket. No other payments or rewards are available.
The need for DMC members to disclose information about any competing interests	To ensure the credibility of the trial, all members of the DMC should disclose any competing interests. These are not restricted to financial matters – involvement in other trials or intellectual investment could be relevant. Although members may well be able to act objectively despite such connections, complete disclosure enhances credibility. (See Annexe 1)<br>DMC members should not use interim results to inform any media outlets or trading in pharmaceutical shares, and careful consideration should be given to trading in stock of companies with competing products.
**6. Organisation of meetings**	
Expected frequency of DMC meetings	The exact frequency of meetings will depend upon any statistical plans specified and on trial events. The wishes of the DMC and needs of the trial office will be considered when planning each meeting.<br>At the end of the feasibility study, the DMC will be convened and a report providing data on the progression targets will be provided. The targets are set out in the protocol.<br>Should the trial continues the DMC will then meet at least annually.

CONTENT	CHARTER DETAILS
Whether meetings will be face-to-face or by teleconference	The first meeting should ideally be face-to-face to facilitate full discussion and allow members to get to know each other. Subsequent meetings will likely be by teleconference or videoconference, but face-to-face meeting may be arranged if felt appropriate.
How DMC meetings will be organised, especially regarding open and closed sessions, including who will be present in each session	A mixture of open and closed sessions is recommended.<br>A closed session is one where only DMC members and others whom they specifically invite, e.g. Trial Statistician, are present.<br>An open sessions is one where all those attending the closed session may be joined by the CI, other members of the trial team and sometimes also representatives of the sponsor, funder, or regulator, as relevant.<br><br>The format of the meetings will be based on the following structure:<br>1. Open session: Introduction and any "open" parts of the report.<br>2. Closed session: DMC discussion of "closed" parts of the report and, if necessary, the trial statistician will attend only part of these discussions.<br>3. Open session: Discussion with other attendees on any matters arising from the closed session.<br>4. Closed session: extra closed session (if necessary).
**7. Trial documentation and procedures to ensure confidentiality and proper communication**	
Intended content of material to be available in open sessions	Open Sessions: Accumulating information relating to recruitment and data quality (e.g. data return rates, treatment adherence) will be presented. Toxicity details based on pooled data will be presented and primary outcome measure data and other outcome measures data may be presented, at the discretion of the DMC.
Intended content of material to be available in closed sessions	Closed sessions: In addition to all the material available in the open session, the closed session material will include efficacy and safety data by procedure.
Whether or not the DMC will be blinded to the treatment allocation	The DMC will be blinded to procedure allocation, with data presented in the closed report as Procedure A and Procedure B. The DMC can ask to be unblinded to procedure allocation should the committee feel they need to know the arms for safety reasons.
The people who will see the accumulating data and interim analysis	Only the members of the DMC (and the statisticians who supply the confidential analyses) will see the accumulating data and interim analyses. The TSC, TMG and the investigators will only be made aware of the interim results if new evidence emerges from other sources or if the trial provides "proof beyond reasonable doubt".<br>DMC members do not have the right to share confidential information with anyone outside the DMC, including the PI.
Responsibility for identifying and circulating external evidence (e.g. from other trials/ systematic reviews)	Identification and circulation of external evidence (e.g. from other trials/ systematic reviews) is not the responsibility of the DMC members. The CI and the trial team will collate any such information for presentation in an open session.

CONTENT	CHARTER DETAILS
To whom the DMC will communicate the decisions/ recommendations that are reached	The DMC reports its recommendations in writing to the TSC. This should be copied to the Trial Statistician and Trial Manager and if possible should be sent via the trial office in time for consideration at a TSC meeting.<br>If the trial is to continue largely unchanged, it is useful for the report from the DMC to include a summary paragraph suitable for trial promotion purposes i.e. to be circulated to trial sites (See Annexe 3).
Whether reports to the DMC be available before the meeting or only at/during the meeting	The trial statistician will aim to prepare the report for the DMC at least 2 weeks before any meetings. Depending on the trial and the nature of the information, it may sometimes be preferable for all papers to be brought to face-to-face meetings by the trial statistician; time would then be needed for DMC members to assimilate the report.
What will happen to the confidential papers after the meeting	The DMC members should store securely the papers after each meeting so they may check the next report against them. After the trial is reported, the DMC members should destroy all interim reports.
**8. Decision making**	
What decisions/recommendations will be open to the DMC	Possible recommendations from the DMC include:-<br>• No action needed, trial continues as planned<br>• Early stopping due, for example, to clear benefit or harm of a treatment, futility or external evidence.<br>• Stopping recruitment within a subgroup (care should be taken if this is not a pre-specified subgroup).<br>• Extending recruitment (based on actual control arm response rates being different to predicted rather than on emerging differences)[2] or follow-up<br>• Proposing or commenting on proposed protocol changes
The role of formal statistical methods, specifically which methods will be used and whether they will be used as guidelines or rules	Following the internal pilot phase, it is anticipated that interim analyses will occur at 12-monthly intervals for review by the DMC, but this could occur more frequently if requested by the DMC members.<br>Formal statistical methods will be used as guidelines rather than absolute rules with respect to the possible recommendations listed above. This is because they generally only consider one dimension of the trial. Reasons should be recorded for disregarding a stopping guideline.<br>The DMC should consider whether any of the randomised comparisons in the trial have provided both (a) “proof beyond reasonable doubt” that for all, or some, types of patient one particular treatment is definitely indicated or definitely contraindicated in terms of a net difference in the major endpoints, and (b) evidence that might reasonably be expected to influence the patient management of many clinicians who are already aware of the other main trial results.<br>*“Proof beyond reasonable doubt” cannot be specified precisely, but a difference of at least*

[2] See concerns on in Section 2

CONTENT	CHARTER DETAILS
	p<0.001 (similar to the Haybittle-Peto stopping boundary) in an interim analysis of a major endpoint may be needed to justify halting, or modifying, the study prematurely. If this criterion were to be adopted, it would have the practical advantage that the exact number of interim analyses would be of little importance, so no fixed schedule is proposed.
How decisions or recommendations will be reached within the DMC	Decisions or recommendations from the DMC will be reached following discussion. The Chair is to summarise discussions and encourage consensus. To aid this it may be best for the Chair to give their own opinion last.<br>It is recommended that every effort should be made for the DMC to reach a unanimous decision. If the DMC cannot achieve this, a vote may be taken, although details of the vote should not be routinely included in the report to the TSC as these may inappropriately convey information about the state of the trial data.<br>It is important that the implications (e.g. ethical, statistical, practical, financial) for the trial be considered before any recommendation is made.
When the DMC is quorate for decision-making	Efforts should be made to ensure that all members can attend. The trial team will try to ensure that a date is chosen to enable this. Members who cannot attend in person should be encouraged to attend by teleconference. If, at short notice, any DMC members cannot attend at all then the DMC may still meet if at least one statistician and one clinician, including the Chair (unless otherwise agreed), will be present. If the DMC is considering recommending major action after such a meeting the DMC Chair should communicate with the absent members as soon after the meeting as possible to check they agree with the proposed action. If they do not, a further meeting should be arranged with the full DMC.
Can DMC members who cannot attend the meeting input	If the report is circulated before the meeting, DMC members who will not be able to attend the meeting may pass comments to the DMC Chair for consideration during the discussions.
What happens to members who do not attend meetings	If a member does not attend a meeting, it should be ensured that the member is available for the next meeting. If a member does not attend the following meeting, they should be asked if they wish to remain part of the DMC. If a member does not attend a third meeting, they should be replaced.
Whether different weight will be given to different endpoints (e.g. safety/efficacy)	There will be no different weight given to different endpoints in the trial.
Any specific issues relating to the trial design that might influence the proceedings, e.g. cluster trials, equivalence trials, multi-arm trials	There are no such issues in the trial.
**9. Reporting**	
To whom will the DMC report their	This will be through a letter to the TSC via the trial statistician, usually

CONTENT	CHARTER DETAILS
recommendations/decisions, and in what form	within 2 weeks of the meeting. A copy of this will be stored at the trial office.
Whether minutes of the meeting be made and, if so, by whom and where they will be kept	Separate records will be required for open and closed sessions with minutes made by the appropriate attending member of the trial team, e.g. Trial Manager or Trial Statistician. Closed session minutes should be stored securely. The DMC Chair should sign off any minutes or notes.
What will be done if there is disagreement between the DMC and the body to which it reports	If the DMC has serious problems or concerns with the TSC decision a meeting of these groups should be held. The information to be shown would depend upon the action proposed and the DMC's concerns. Depending on the reason for the disagreement confidential data would often have to be revealed to all those attending such a meeting. The meeting would be Chaired by a senior member of the BCTU staff or an external expert who is not directly involved with the trial.
**10. After the trial**	
Publication of results	At the end of the trial there may be a meeting to allow the DMC to discuss the final data with the writing committee to give advice about data interpretation.<br>The DMC may wish to see a statement that the trial results will be published in a correct and timely manner.
The information about the DMC that will be included in published trial reports	DMC members will be named and their affiliations listed in the main report, unless they explicitly request otherwise. A brief summary of the timings and conclusions of DMC meetings should be included in the body of this paper.
Whether the DMC will have the opportunity to approve publications, especially with respect to reporting of any DMC recommendation regarding termination of a trial	The DMC may wish to be given the opportunity to read and comment on publications before submission.
Any constraints on DMC members divulging information about their deliberations after the trial has been published	Members of the DMC should not discuss any issues that arise from their involvement in the trial for at least 12 months after the primary trial results have been published, or when permission is agreed with the overseeing committee.

Abbreviations and glossary

BCTU	Birmingham Clinical Trials Unit
CI	Chief Investigator
DMC	Data Monitoring Committee
EUDRACT	European Union Drug Regulatory Agency Clinical Trial
ISRCTN	International standard randomised controlled trial number
SAE	Serious adverse event
TMG	Trial Management Group
TSC	Trial Steering Committee

Figure 1: Relationship of trial committees

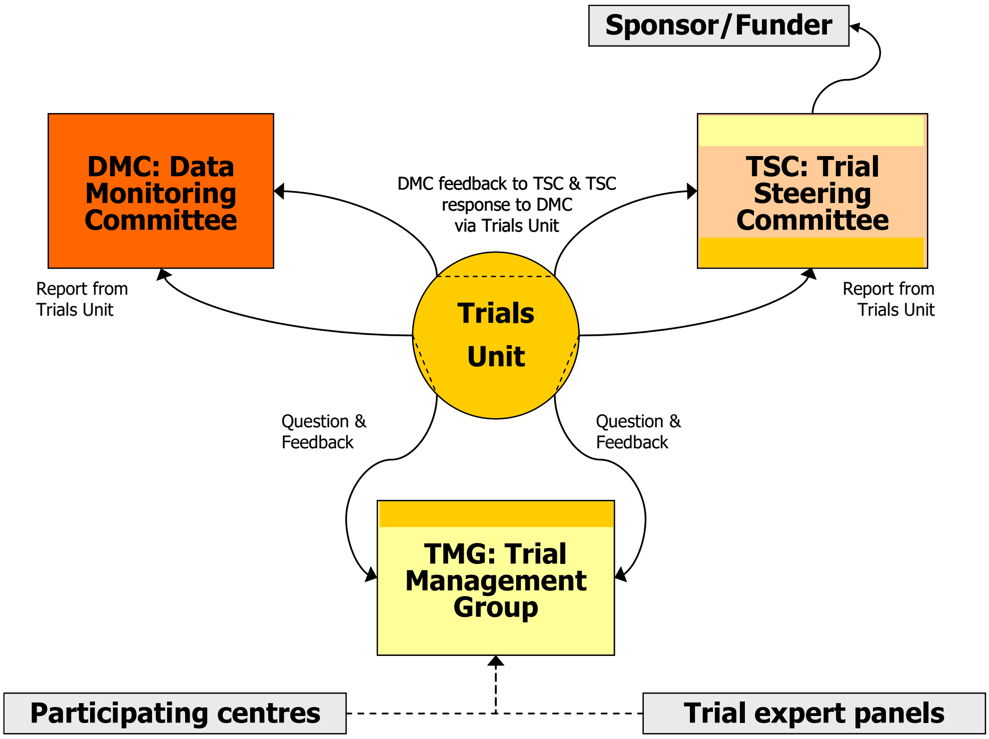

Annexe 1: Agreement and potential competing interests form

<u>ADEPP Data Monitoring Committee:</u> Agreement to join the Data Monitoring Committee and disclosure of potential competing interests

Please complete the following document and return to the Trial Manager.

(please initial box to agree)

☐	I have read and understood the DMC Charter version……… dated………………………………
☐	I agree to join the Data Monitoring Committee for this trial
☐	I agree to treat all sensitive trial data and discussions confidentially

The avoidance of any perception that members of a DMC may be biased in some fashion is important for the credibility of the decisions made by the DMC and for the integrity of the trial.

Possible competing interest should be disclosed via the BCTU. In many cases simple disclosure up front should be sufficient. Otherwise, the (potential) DMC member should remove the conflict or stop participating in the DMC. **Table 1** lists potential competing interests.

☐ **No,** I have no competing interests to declare
☐ **Yes,** I have competing interests to declare (please detail below)

Please provide details of any competing interests:

____

____

____

Name: ____________________________

Signed: ___________________________ Date: ______________

Table 1: Potential competing interests

- Stock ownership in any commercial companies involved
- Stock transaction in any commercial company involved (if previously holding stock)
- Consulting arrangements with the Sponsor (including CI for other trials)
- Frequent speaking engagements on behalf of the intervention
- Career tied up in a product or technique assessed by trial
- Hands-on participation in the trial
- Involvement in the running of the trial
- Emotional involvement in the trial
- Intellectual conflict e.g. strong prior belief in the trial's experimental arm
- Involvement in regulatory issues relevant to the trial procedures
- Investment (financial or intellectual) or career tied up in competing products
- Involvement in the publication in the form of authorship

Note: This DMC template was developed using on MRC CTU template DMC Charter v2.01, 13-Mar-2006; from DAMOCLES DMC Charter template v1, Feb 2005

Annexe 2: Agreement and confidentiality agreement for observers

<u>ADEPP Data Monitoring Committee</u>: Agreement to attend the Data Monitoring Committee and treat all information confidentially

Please complete the following document and return to the Trial Manager.

(please initial box to agree)

☐	I have received a copy of the DMC Charter version……… dated………………………………..
☐	I agree to treat as confidential any sensitive trial information gained during this meeting unless explicitly permitted

Name: ___________________________

Signed: __________________________ Date: ______________

Note: This DMC template was developed using on MRC Clinical Trials Unit Cancer Group DMC Charter template v2.01, 13-Mar-2006; from DAMOCLES DMC Charter template v1, Feb 2005

Annexe 3: Suggested report from DMC to TSC where no recommendations are being made

[Insert date]

To: Chair of Trial Steering Committee
Via: Trial Manager

Dear *[Chair of Trial Steering Committee]*

The Independent Data Monitoring Committee (DMC) for the *[insert trial name]* trial met on *[meeting date]* to review its progress and interim accumulating data. *[List members] attended* the meeting and reviewed the report.

The DMC should like to congratulate the investigators and trial team on the running of the trial and its recruitment, data quality and follow-up. The trial question remains important and, on the basis of the data reviewed at this stage, we recommend continuation of the trial according to the current version of the protocol *[specify protocol version number and date]* with no changes.

We shall next review the progress and data *[provide approximate timing]*

Yours sincerely,

*[*Name of meeting Chair*]*
Chair of Independent Data Monitoring Committee

On behalf of the DMC (all members listed below)

DMC members:
(1) [*Insert name and role*]
(2) [*Insert name and role*]
(3) [*Insert name and role*]
(4) [*Insert name and role*]
